# Incorporation of albumin fusion proteins into fibrin clots *in vitro *and *in vivo*: comparison of different fusion motifs recognized by factor XIIIa

**DOI:** 10.1186/1472-6750-11-127

**Published:** 2011-12-20

**Authors:** William P Sheffield, Louise J Eltringham-Smith

**Affiliations:** 1 Department of Pathology and Molecular Medicine, McMaster University, 1200 Main Street West, Hamilton, Ontario, Canada; 2 Research and Development, Canadian Blood Services, Hamilton, Ontario, Canada

## Abstract

**Background:**

The transglutaminase activated factor XIII (FXIIIa) acts to strengthen pathological fibrin clots and to slow their dissolution, in part by crosslinking active α_2_-antiplasmin (α_2_AP) to fibrin. We previously reported that a yeast-derived recombinant fusion protein comprising α_2_AP residues 13-42 linked to human serum albumin (HSA) weakened *in vitro *clots but failed to become specifically incorporated into *in vivo *clots. In this study, our aims were to improve both the stability and clot localization of the HSA fusion protein by replacing α_2_AP residues 13-42 with shorter sequences recognized more effectively by FXIIIa.

**Results:**

Expression plasmids were prepared encoding recombinant HSA with the following N-terminal 23 residue extensions: H_6_NQEQVSPLTLLAG_4_Y (designated XL1); H_6_DQMMLPWAVTLG_4_Y (XL2); H_6_WQHKIDLPYNGAG_4_Y (XL3); and their 17 residue non-His-tagged equivalents (XL4, XL5, and XL6). The HSA moiety of XL4- to XL6-HSA proteins was C-terminally His-tagged. All chimerae were efficiently secreted from transformed *Pichia pastoris *yeast except XL3-HSA, and following nickel chelate affinity purification were found to be intact by amino acid sequencing, as was an N-terminally His-tagged version of α_2_AP(13-42)-HSA. Of the proteins tested, XL5-HSA was cross-linked to biotin pentylamine (BPA) most rapidly by FXIIIa, and was the most effective competitor of α_2_AP crosslinking not only to BPA but also to plasma fibrin clots. In the mouse ferric chloride *vena cava *thrombosis model, radiolabeled XL5-HSA was retained in the clot to a greater extent than recombinant HSA. In the rabbit jugular vein stasis thrombosis model, XL5-HSA was also retained in the clot, in a urea-insensitive manner indicative of crosslinking to fibrin, to a greater extent than recombinant HSA.

**Conclusions:**

Fusion protein XL5-HSA (DQMMLPWAVTLG_4_Y-HSAH_6_) was found to be more active as a substrate for FXIIIa-mediated transamidation than seven other candidate fusion proteins *in vitro*. The improved stability and reactivity of this chimeric protein was further evidenced by its incorporation into *in vivo *clots formed in thrombosis models in both mice and rabbits.

## Background

Recombinant serum albumins provide an attractive scaffold for the delivery of attached therapeutic peptides or proteins [1,2]. They are the most abundant proteins in the plasma of mammals, reaching concentrations of 40-80 mg/mL (0.6-1.0 mM) in humans [3]. They are slowly cleared plasma proteins [3,4] whose longevity in the circulation derives from a well-characterized mechanism of recycling via the major histocompatibility complex-related Fc receptor for immunoglobulin G (FcRn) [5-7]. Serum albumin is also unusual among plasma proteins in that it is not glycosylated [3], a property that simplifies its recombinant expression and production [8]. If candidate therapeutic peptides or proteins can be fused to albumin in a manner that allows them to express their endogenous activity, the resulting fusion protein often demonstrates improved pharmacokinetic and pharmacodynamic properties due to acquisition of albumin-like circulatory characteristics [1]. For these reasons, albumin has been fused to numerous proteins such as interferon [9], interleukin-2 [10], butrylcholinesterase [11], coagulation factors VII [12] and IX [13,14], hirudin [15,16], and barbourin [17], as well as to smaller peptides [18].

In order to address unmet clinical needs in the area of thrombosis, the pathological formation of clots within intact blood vessels, our laboratory previously investigated novel recombinant proteins comprised of portions of α_2_-antiplasmin fused to the N-terminus of human serum albumin [19]. α_2_AP is cross-linked to fibrin clots by activated factor XIII (FXIIIa), where it promotes clot stability by inhibiting the fibrinolytic enzyme plasmin [20,21]. We [19] and others [22,23] have demonstrated that competing α_2_AP-fibrin crosslinking results in fibrin clots that are more easily dissolved *in vitro*, suggesting potential applications of such competitors as adjuncts to thrombolytic therapy.

Previously, we attempted expression of three α_2_AP-HSA fusion proteins in *Pichia pastoris*, a methylotropic yeast shown to produce recombinant HSA in high yield and with indistinguishable biophysical properties to its plasma-derived counterpart [24]. Our strategy was to retain the N-terminal sites of α_2_AP-fibrin crosslinking, especially Q14 [25], but to delete the C-terminal plasmin-binding and plasmin-trapping reactive centre loop of this serpin-type inhibitor. We sought to provide as native a protein environment as possible for the α_2_AP cross-linking motif by maximizing the portion of α_2_AP fused to HSA. However, only a chimeric protein comprised of α_2_AP residues 13-42 fused to HSA was secreted by the yeast; the two longer fusions involving residues 13-73 and 13-109 were not stably expressed. Moreover, α_2_AP(13-42)-HSA was partially proteolyzed within its α_2_AP moiety. While we demonstrated that α_2_AP(13-42)-HSA became a substrate of FXIIIa and competed for α_2_AP crosslinking to both small and macromolecular substrates, we could not demonstrate cross-linking to clots *in vivo *above background levels seen with recombinant HSA [19].

In the present study, we sought to improve the ability of HSA fusion proteins to compete with α_2_AP for FXIIIa-mediated cross-linking. The α_2_AP moiety was reduced to 12 residues (α_2_AP 13-23 followed by Lys24Ala), to avoid previously observed sites of proteolysis by *P. pastoris *proteases, and compared to two 12-residue artificial FXIIIa substrate peptides selected by phage display for high reactivity [26]. These motifs were expressed with or without N-terminal His tags, to allow for purification of full length proteins away from partially proteolyzed products. We hypothesized that replacing α_2_AP 13-42 with shorter, potentially more active FXIIIa substrate motifs would improve the stability of fibrin cross-linkable HSA proteins and improve *in vivo *clot retention.

## Methods

### DNA Manipulations

All oligonucleotides employed in this study were synthesized at MOBIX Lab, a McMaster University core facility. The sequences of all plasmids generated in this study were validated by DNA sequencing, also performed by MOBIX Lab, prior to use for protein expression. Standard molecular biological protocols for DNA restriction and ligation, plasmid mini-DNA preparation, purification of DNA from agarose gels, and transformation of *E. coli *Top10 (Invitrogen) to ampicillin resistance, were employed [19]. Three plasmids encoding fusion proteins XL4-, XL5-, and XL6-HSA were first prepared, by analogous means. Synthetic oligonucleotides, whose sequences are shown in Table 1, were first annealed by heating to 95°C and slow cooling to room temperature, in pairs, for each construct, respectively: ML-08-5116 and ML-08-5117; ML-08-5118 and ML-08-5119; and ML-08-5120 and ML-ML-08-5121. Annealed oligonucleotides were then separately ligated to the 6325 bp XhoI-KpnI restriction digestion fragment of previously described plasmid pPICZ9ss-α_2_AP(13-73)-HSAH_6 _[19], replacing the α_2_AP(13-73) codons, forming plasmids pPICZ9ss-XL#-HSAH_6_, where # corresponds to 4, 5, or 6, respectively. In order to construct XL-HSA constructs in which the His tag was not present on the C-terminus of the HSA moiety, an intermediate construct was prepared. A full-length preproHSA cDNA in pCDNA3.1 (Invitrogen), pC3ppHSA, was restricted with XbaI and ApaI, and the 804 bp minor fragment was ligated to the major 5786 bp XbaI-ApaI fragment of pPICZ9ss-α_2_AP(13-73)-HSAH_6_, yielding plasmid pPICZ9ss-α_2_AP(13-73)-HSA, in which the HSA open reading frame terminated without hexahistidine codons. The 5768 bp XhoI-KpnI restriction fragment of pPICZ9ss-α_2_AP(13-73)-HSA was then combined with the following pair-wise annealed oligonucleotides: ML-08-5104 and ML-08-5105; ML-08-5106 and ML-08-5107; and ML-08-5108 and ML-08-5109. This approach yielded plasmids pPICZ9ss-XL#-HSAH_6_, where # corresponds to 1, 2, or 3, respectively. A final expression plasmid was constructed to reverse the orientation of the His tag of fusion protein α_2_AP(13-73)-HSAH_6 _[19]. Plasmid pBAD-H_6_-α_2_AP [27] was PCR-amplified using oligonucleotides ML 09-3874 and 17225 [27], and the 125 bp XhoI-KpnI restriction fragment of the resulting PCR product was inserted between the corresponding sites of pPICZ9ss-α_2_AP(13-73)-HSA to yield pPICZ9ss-H_6_α_2_AP(13-42)-HSA. Each completed plasmid listed above was used to transform *Pichia pastoris *strain X33 to Zeocin (Invitrogen) resistance as previously described [18].

**Table 1:** Oligonucleotides employed in this study

Oligonucleotide		Size	Purpose
**Number**	Sequence	(nt)	

**Ml-08-5104**	5'-TCGAGAAAAG ACATCATCAT CATCATCATA ACCAGGAGCA GGTGTCCCCA CTTACCCTCC TCGCTGGAGG TGGAGGGTAC-3'	80	XhoI-KpnI sense strand for XL1-HSA

**ML-08-5105**	5'-CCTCCACCTC CAGCGAGGAG GGTAAGTGGG GACACCTGCT CCTGGTTATG ATGATGATGA TGATGTCTTT TC-3'	72	XhoI-KpnI antisense strand for XL1-HSA

**ML-08-5106**	5- TCGAGAAAAG ACATCATCAT CATCATCATG ATCAGATGAT GCTGCCATGG CCAGCTGTGA CCCTGGGAGG CGGAGGGTAC -3'	80	XhoI-KpnI sense strand for XL2-HSA

**ML-08-5107**	5- CCTCCGCCTC CCAGGGTCAC AGCTGGCCAT GGCAGCATCA TCTGATCATG ATGATGATGA TGATGTCTTT TC-3'	72	XhoI-KpnI antisense strand for XL2-HSA

**ML-08-5108**	5'-TCGAGAAAAG ACATCATCAT CATCATCATT GGCAGCATAA AATCGATCTG CCATACAATG GTGCAGGAGG CGGAGGGTAC-3'	80	XhoI-KpnI sense strand for XL3-HSA

**ML-08-5109**	5'- CCTCCGCCTC CTGCACCATT GTATGGCAGA TCGATTTTAT GCTGCCAATG ATGATGATGA TGATGTCTTT TC-3'	72	XhoI-KpnI antisense strand for XL3-HSA

**ML-08-5116**	5'- TCGAGAAAAG AAACCAGGAG CAGGTGTCCC CACTTACCCT CCTCGCTGGA GGTGGAGGGT AC -3'	62	XhoI-KpnI sense strand for XL4-HSA

**ML-08-5117**	5'- CCTCCACCTC CAGCGAGGAG GGTAAGTGGG GACACCTGCT CCTGGTTTCT TTTC-3'.	54	XhoI-KpnI antisense strand for XL4-HSA

**ML-08-5118**	5'- TCGAGAAAAG AGATCAGATG ATGCTGCCAT GGCCAGCTGT GACCCTGGGA GGCGGAGGGT AC -3'	62	XhoI-KpnI sense strand for XL5-HSA

**ML-08-5119**	5'- CCTCCGCCTC CCAGGGTCAC AGCTGGCCAT GGCAGCATCA TCTGATCTCT TTTC -3'	54	XhoI-KpnI antisense strand for XL5-HSA

**ML-08-5120**	5'-TCGAGAAAAG ATGGCAGCAT AAAATCGATC TGCCATACAA TGGTGCAGGA GGCGGAGGGT AC-3'	62	XhoI-KpnI sense strand for XL6-HSA

**ML-08-5121**	5'-CCTCCGCCTC CTGCACCATT GTATGGCAGA TCGATTTTAT GCTGCCATCT TTTC-3'	54	XhoI-KpnI antisense strand for XL6-HSA

**ML-09-3874**	5'-ACGTGTCGAGA AAAGACATCA TCATCATCAT CATAACCAGG AGCAGGTGTC CCCAC-3'	45	Upstream primer for PCR to assemble H_6_α_2_AP(13-42)-HSA

**ML 17225**	5'-ACGTGGTACCG ACTCCTGGGG GACTCTTCAG-3'	30	Downstream primer for PCR to assemble H_6_α_2_AP(13-42)-HSA

### Fusion protein expression, purification, and characterization

HSA fusion proteins were purified from media conditioned by transformed *Pichia pastoris *cells and induced with 0.5% vol/vol methanol, as previously described [18,19,28], except that the methanol induction phase of protein production was extended to 96 hours. At that point, the conditioned media was neutralized and clarified by centrifugation [1], treated with protease inhibitors (5 mM benzamidine and 0.1 mM phenylmethylsulfonyl fluoride), and purified using Ni-NTA agarose chromatography (Qiagen). Purified proteins were analyzed by SDS-PAGE and immunoblotting using polyclonal affinity-purified goat anti-human α_2_AP antibodies (Affinity Biologicals) and murine monoclonal anti-HSA antibodies (Genway Biotech). They were also characterized by automated Edman degradation at the Advanced Protein Technology Centre of the Hospital for Sick Children, Toronto, Canada.

### Transglutamination assays with biotinylated pentylamine

In order to quantify FXIIIa-catalyzed transglutamination of plasma-derived α_2_AP and recombinant HSA fusion proteins, a microtiter plate (Immulon 4 HBX, Thermo Scientific) protocol was developed. Test HSA-related proteins (1.7 μM) were first incubated for varying times in Tris-buffered saline (50 mM Tris-Cl pH 7.5, 150 mM NaCl) containing 5 mM CaCl_2 _and 5 mM biotinylated pentylamine (BPA; EZ-Link pentylamine-biotin, Pierce) supplemented with 20 nM human FXIII (Enzyme Research Labs, ERL) and 1.0 IU/mL human thrombin (ERL). Reactions (0.1 mL) were stopped by addition of an equal volume of 20 mM EDTA pH 8.00 and transferred to microtiter plates. Microtiter plates were prepared for use by coating with 2.5 μg/mL mouse anti-HSA monoclonal antibody (Genway) in 50 mM sodium carbonate pH 9.6 overnight at 4°C. All other incubations were at room temperature. All washes were performed with Tris-buffered saline supplemented with 0.05% (vol/vol) Tween 20 (TBST), and repeated three times. Anti-HSA-coated plates were washed and then blocked for one hour in TBST, then washed again. Following transfer of the stopped transglutamination reaction mixtures to the plate, it was incubated with shaking for one hour and washed, prior to reaction of captured proteins with a 1:2500 dilution of alkaline phosphatase-conjugated streptavidin (Jackson Labs) in TBST for one hour. Following a final wash step, colour was developed by addition of 1.0 mg/ml *p*-nitrophenyl phosphate disodium salt (PNPP) in diethanolamine buffer (1.02 M diethanolamine pH 9.8) (Thermo Scientific) and absorbance was quantified on an ELx808 plate reader (BioTek Instruments) at 405 nm for up to 15 minutes. In some reactions, the protocol was modified to measure transglutamination of α_2_AP by substituting anti-α_2_AP antibodies for anti-HSA antibodies, and purified human plasma-derived α_2_AP (ERL) for HSA-related proteins.

### Transglutamination of fibrin(ogen)

FXIIIa-catalyzed transglutamination of the natural substrate fibrinogen was assessed using SDS-PAGE and immunoblotting of cross-linking reactions as previously described [19]. Briefly, 100 nM FXIII was first activated to FXIIIa by reaction with 5.0 IU/ml thrombin for 5 minutes at 37°C; thrombin was then inactivated using Phe-Pro-Arg chloromethylketone to 10 μM final concentration, to prevent clotting on addition to fibrinogen. The resulting FXIIIa (50 nM) was reacted with 6.0 μM human fibrinogen (specifically depleted of plasminogen, von Willebrand factor, and fibronectin, ERL), and 1.0 μM substrate proteins in TBS containing 10 mM CaCl_2_.

Reactions were stopped by addition of SDS and analyzed on 8% SDS-polyacrylamide gels under reducing conditions, with immunoblotting and chemiluminescent development of antibody-decorated blots, as described [19].

### *In vitro *clot lysis

As previously described [19], the formation and dissolution of human plasma clots was followed by recording changes in turbidity, using an ELx808 plate reader (BioTek Instruments) set to take absorbance readings at 340 nm every 30 seconds for 4 hours, and quantified as the area under the curve.

### Retention of iodinated proteins in mouse *vena cava *treated with ferric chloride

Purified human fibrinogen (Sigma), human α_2_AP (ERL), or XL5-HSA recombinant proteins were iodinated using the Iodogen method as described by the manufacturer (Pierce), using either sodium ^125^I or ^131^I. Unincorporated radioactivity was removed by exhaustive dialysis versus phosphate-buffered saline. Specific activities of labelling exceeded 1 × 10^9 ^cpm/mg. Radioiodinated proteins were then tested in the mouse ferric chloride *vena cava *thrombosis model, modified from [29,30]. CD-1 mice were anaesthetized using gaseous anaesthesia (3% isofluorane) at all times during this procedure, and a heating pad was employed to ensure maintenance of normal body temperature. A mid-line incision was made, first through the skin and secondly through the muscle layer. Viscera were gently displaced and haemostatic clamps employed to keep the incision open. At this time radioiodinated proteins (5 × 10^6 ^cpm in 0.1 ml sterile saline) were injected via the tail vein. The *vena cava *was then exposed and a 2 × 4 mm piece of Whatman paper soaked in 10% (w/vol) ferric chloride was applied. The viscera were covered with gauze soaked in warm saline during this time. The time from radioactive protein injection to application of ferric chloride was fixed at 5 minutes. Three minutes after its application, the filter paper was removed and viscera replaced. Thirty minutes later, the *vena cava *was re-exposed, the vessel was excised, and the clot was transferred to a tared tube for weighing, and then subjected to γ counting using either an Auto Gamma 5530 Minaxi γ counter (Perkin Elmer) or a Cobra II γ counter (Packard) to quantify incorporated radioactivity.

### Retention of iodinated proteins in rabbit jugular vein thrombi

New Zealand White rabbits were subjected to a modified Wessler procedure [31] as previously described by this laboratory [19]. Briefly, animals were initially anesthetized with 100 mg of ketamine, then maintained in the anesthetized state with 1.5% isoflurane. They were then cannulated via the carotid artery and the jugular veins isolated. One ml of whole blood was freshly drawn and anticoagulated with 1/9^th ^volume of 3.8% w/vol sodium citrate. Two centimeter long sections of the right and left jugular veins were isolated, emptied of blood, and isolated using bulldog clamps. Clotting of the anticoagulated, autologous whole blood was initiated by combining it with warm (37°) human thromboplastin reagent Thromborel S (Dade Behring) in a 1:4 (vol:vol) ratio, supplementing it to 3.3 × 10^6 ^cpm/ml ^131^I- fibrinogen and ^125^I-recombinant protein, and re-introducing 0.15 ml of the clotting blood into the isolated segments. This was done for both isolated jugular veins and blood flow was held in stasis for 30 minutes, at which time the clamps were removed, and blood flow restored. Clots were recovered 60 minutes later, following jugular vein opening by incision, and removed, weighed, and γ-counted as described above for murine clots. In some experiments clots were extracted overnight in 5.0 M urea, microcentrifuged, and the supernatant removed prior to re-counting. The proportion of urea-stable protein incorporation was then calculated, adjusted for radioactive decay between the recorded times of the first and second γ-count. All animal experiments were carried out under the terms of an approved Animal Utilization Protocol reviewed, approved, and monitored by the Animal Research Ethics Board of the Faculty of Health Sciences, McMaster University.

### Statistical analysis

Statistical tests were performed using GraphPad Instat version 4 (GraphPad Software). Multiple comparisons used one-way parametric analysis of variation (ANOVA) with Tukey-Kramer post-tests where data were normally distributed, and nonparametic ANOVA with Dunn's post-tests where they were not.

## Results

### Expression and characterization of fusion proteins

**Figure 1:**
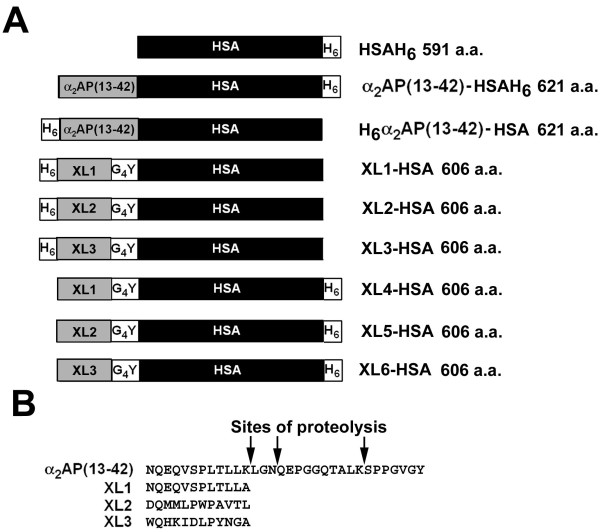
**HSA fusion protein design, expression, and characterization**. (A) Schematic diagram of proteins. Relevant polypeptides are shown in linear form, oriented from N-terminus to C-terminus, with different regions labelled on the bar diagram. HSA residues are shown in black, while candidate FXIIIa substrate motifs are shown in grey. White bars correspond either to hexahistidine tags (H_6_) or to tetraglycine-tyrosyl spacer segments (G_4_Y). Polypeptide names and predicted length in amino acids (a.a.) is shown at right. (B) Amino acid sequence of candidate FXIIIa substrate motifs. The amino-terminal portion of α_2_AP previously fused to HSA [19] is shown, including putative sites of proteolysis deduced by amino acid sequencing, on the top line (α_2_AP(13-42)). The three candidate FXIIIa motifs fused to albumin with or without adjacent His tags, are shown below (XL1,2, or 3).

**Figure 2:**
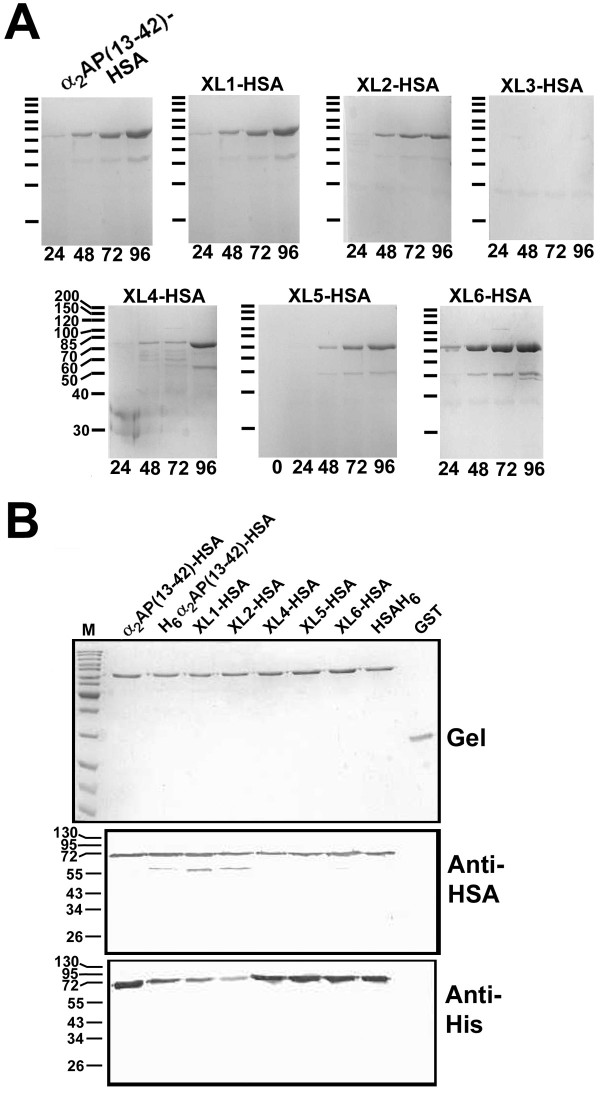
**Expression, purification and characterization of HSA-related recombinant proteins**. (A) Electrophoresis of conditioned media from *P. pastoris *transformed with different expression plasmids. Conditioned media samples taken 24, 48, 72, or 96 hours after the start of methanol induction are shown on 10% reducing SDS-polyacrylamide gels stained with Coomassie Blue. The protein product encoded by the expression plasmid is shown above each gel. The position of molecular mass markers (200, 150, 120,. 100, 85, 70, 60, 50, 40, and 30 kDa) is shown to the left of each panel and labelled (XL4-HSA) in one instance as an example. (B) Upper panel, gel profile of purified recombinant proteins on 10% SDS-polyacrylamide gels electrophoresed under reducing conditions and stained with Coomassie Blue. Proteins (400 ng) are identified above each lane as in Figure 1A, plus GST (*E.coli*-expressed glutathione sulfotransferase); M, molecular mass markers identical to those used in Figure 1A. Middle and lower panel, replicate gels differing only in the amounts of protein loaded per lane, to avoid overloading with antibodies of different affinity, were immunoblotted and probed with Anti-HSA antibodies (middle pane, 100 ng/lane) or anti-hexahistidine antibodies (lower panel, Anti-His, 500 ng/lane).

Media conditioned by *Pichia pastoris *cultures transformed with expression plasmids encoding fusion proteins XL(1-6)-HSA (see Figure 1) were first screened for recombinant expression of HSA-related polypeptides by gel analysis. As shown in Figure 2A, although there were some differences in the time course of expression, five of six of the yeast cell lines secreted a major methanol-dependent polypeptide of the expected size of the HSA fusion proteins, of 70-80 kDa. The exception was the cell line programmed to express XL3-HSA, which secreted no detectable methanol-dependent polypeptides.

The five putative HSA fusion proteins were purified from conditioned media induced with methanol for 96 hours, using nickel chelate affinity chromatography, and reacted both with anti-HSA and anti-hexahistidine antibodies, as shown in Figure 2B. The same properties were observed for H_6_α_2_AP(13-42)-HSA and previously described α_2_AP(13-42)-HSA [19] (Figure 2). Similar results were obtained when an additional two independent *P. pastoris *Zeocin-resistant cell lines were examined, in addition to the ones shown in Figure 2 (data not shown).

Because the minor, 2-4 kDa difference between the shortest (HSAH_6_) and longest (H_6_α_2_AP(13-42)-HSA) putative expression proteins was not resolvable on the gel system we employed, we sought independent confirmation of the identity and integrity of the expressed protein products by amino acid sequencing. For XL(4-6)-HSA, a single N-terminal sequence was detected by Edman degradation: NQEQVS; DQMMLP; and WQHKID, respectively. For XL1-HSA and XL2-HSA, Edman degradation showed intact hexahistidine amino-termini. For H_6_α_2_AP(13-42)-HSA, two sequences were detected: the major sequence arising from the expected hexahistidine tag, and the other a mixture of amino acids suggestive of a similar pattern of partial proteolysis we previously reported for α_2_AP(13-42)-HSA [19]. In that regard, batches of the latter protein were prepared using 96 hours of methanol induction, as employed for all other proteins in this study, for consistency and for comparative purposes. Edman degradation showed a mixture of termini similar to those previously observed from cultures induced for lesser periods of time [19]. These included termini commencing with Leu23 (LKLGNQ), Leu25 (LGNQEP), and Ser38 (SPPGVC) but no detectable full-length product. Three separate batches of such protein preparations returned similar results (data not shown).

While the amino acid sequencing results showed that positioning of a hexahistidine tag on the N-terminus of α_2_AP(13-42)-HSA improved the integrity of the resulting purified protein product, the yield was reduced from 40-50 mg/l for AP(13-42)-HSA to 5-6 mg/l for H_6_α_2_AP(13-42)-HSA. Yields for the purified XL-HSA proteins varied from 10-12 mg/l (XL1- and XL2-HSA) to 20-35 mg/l ( XL4- and XL5-HSA) to 80-85 mg/l (XL6-HSA).

### Characterization of recombinant fusion proteins as fXIIIa substrates for cross-linking to lysine donors

**Figure 3:**
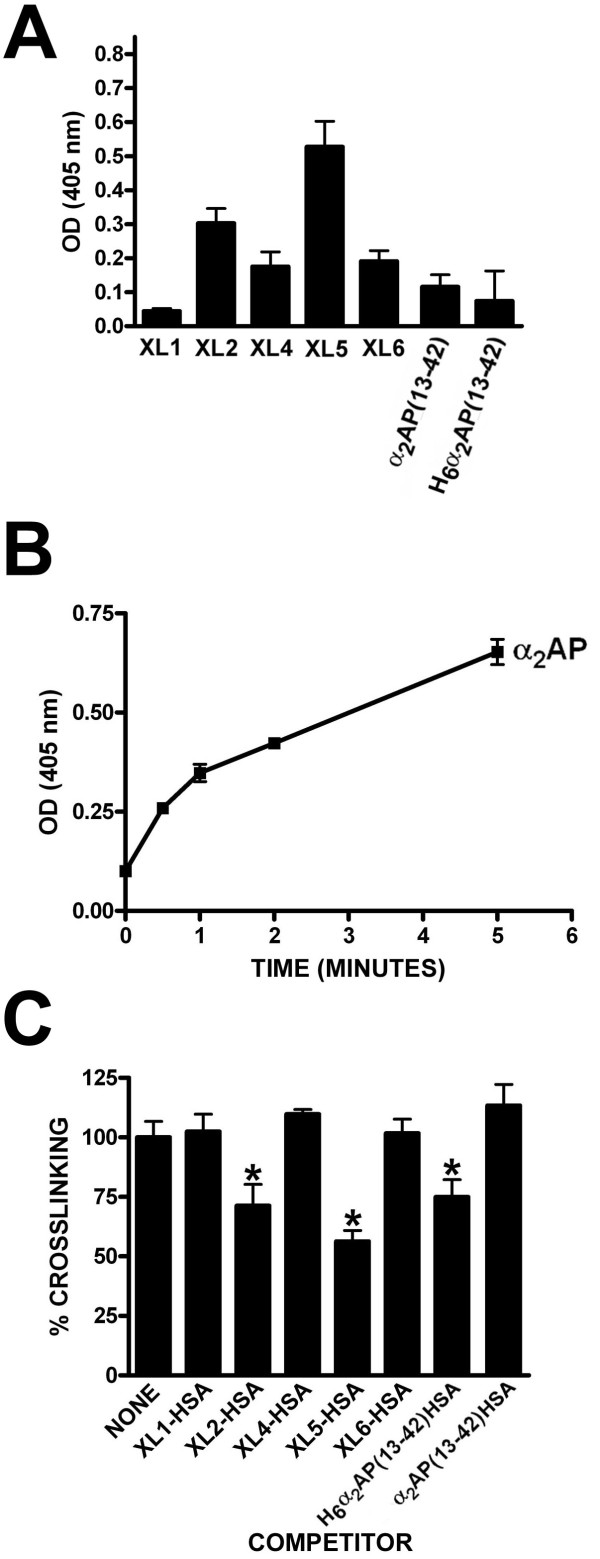
**FXIIIa-mediated cross-linking of recombinant proteins and plasma-derived α**_**2**_**AP to biotinylated pentylamine**. Panel A shows transglutamination reactions, in which 20 nM FXIIIa cross-linked 5 mM BPA to 1.7 μM HSA fusion proteins (identified below the × axis), were stopped after 2.5 minutes and the relative BPA incorporation was quantified using anti-HSA capture and alkaline phosphatase-conjugated streptavidin detection as outlined in "Methods". Colour generation was quantified as the optical density at 405 nm (OD (405 nm)). The mean ± standard deviation (SD) of 6 determinations is shown, with background (reactions lacking FXIIIa) subtracted. Panel B shows a time course of FXIIIa-mediated cross-linking of BPA to 1.7 μM α_2_AP, using the same conditions as employed in Panel A except that anti-α_2_AP antibodies were used to capture biotinylated α_2_AP after reaction termination at various time points. The mean ± the standard deviation (SD) of 3 determinations is shown, with background (reactions lacking FXIIIa) subtracted. Panel C shows BPA-α_2_AP crosslinking reactions of 5 minutes' duration, as shown in panel B, except that competitor proteins identified on the × axis were included at 12 μM. The mean ± the SD of 5-14 determinations is shown, with background (reactions lacking FXIIIa) subtracted.

Having demonstrated that all recombinant fusion proteins with the exception of XL3-HSA had been produced, we next asked whether the addition of the candidate FXIIIa sequences had actually converted the proteins into substrates for cross-linking by FXIIIa. Cross-linking reactions were performed in solution, using the chemical amine donor biotinylated pentylamine, and reaction products were captured using antibodies immobilized on microtiter plate walls and quantified colorimetrically using alkaline-phosphatase-linked streptavidin. As shown in Figure 3A, while there was evidence of some cross-linking for all proteins tested, the highest levels were observed for fusion proteins XL5-HSA and XL2-HSA, in that order. While the results shown in Figure 3A were limited to initial (2.5 minute) reactions, by five minutes the cross-linking of XL2-HSA and XL5-HSA had become comparable (data not shown).

**Figure 4:**
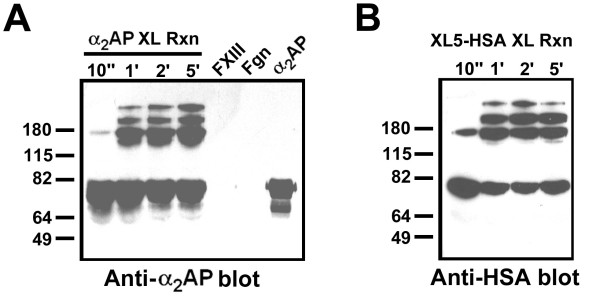
**FXIIIa-mediated cross-linking of α**_**2**_**AP and XL5-HSA to fibrinogen**. FXIII was pre-activated to fXIIIa by thrombin, the thrombin inactivated with FPRck, and the fXIIIa then combined with fibrinogen and α_2_AP (A) or XL5-HSA (B) in transglutamination reactions probed, after reducing SDS-polyacrylamide electrophoresis, with either (A) anti-α_2_AP or (B) anti-HSA antibodies. The position of pre-stained molecular mass markers is indicated to the left of the panels. Reactions containing only FXIII, only fibrinogen, or only α_2_AP are also shown.

Similar results were obtained when HSA fusion proteins were employed as competitors of the FXIIIa-dependent transfer of biotin from BPA to α_2_AP, which exhibited time-dependent crosslinking on the time scale employed (see Figure 3B). As shown in Figure 3C, of seven proteins tested using a seven-fold excess of competitor over α_2_AP, only XL5-HSA, XL2-HSA, and H_6_α_2_AP(13-42)-HSA significantly reduced cross-linking of α_2_AP; the relative efficacy of the proteins was in the order listed. To minimize the possibility that reactivity with BPA was an artefact that did not accurately predict reactivity with macromolecular donors, the ability of XL5-HSA to be cross-linked to fibrinogen was tested. As shown in Figure 4, XL5-HSA and α_2_AP exhibited similar kinetics in forming high molecular weight products with fibrinogen that were dependent on added FXIIIa, although the fact that different antibodies, with potentially different binding efficiency, had to be employed limited the comparison to a qualitative assessment. A similar pattern was observed for both proteins in that three higher molecular weight bands of 140 kDa and greater formed in a factor XIIIa- and fibrinogen-dependent manner; with respect to plasma-derived α_2_AP, these bands have been suggested to represent α_2_AP-fibrinogen Aα chain, α_2_AP-fibrinogen Aα dimer, and α_2_AP-fibrinogen Aα polymers [32].

### Comparison of fusion proteins as competitors of α_2_AP-mediated *in vitro *clot protection

**Figure 5:**
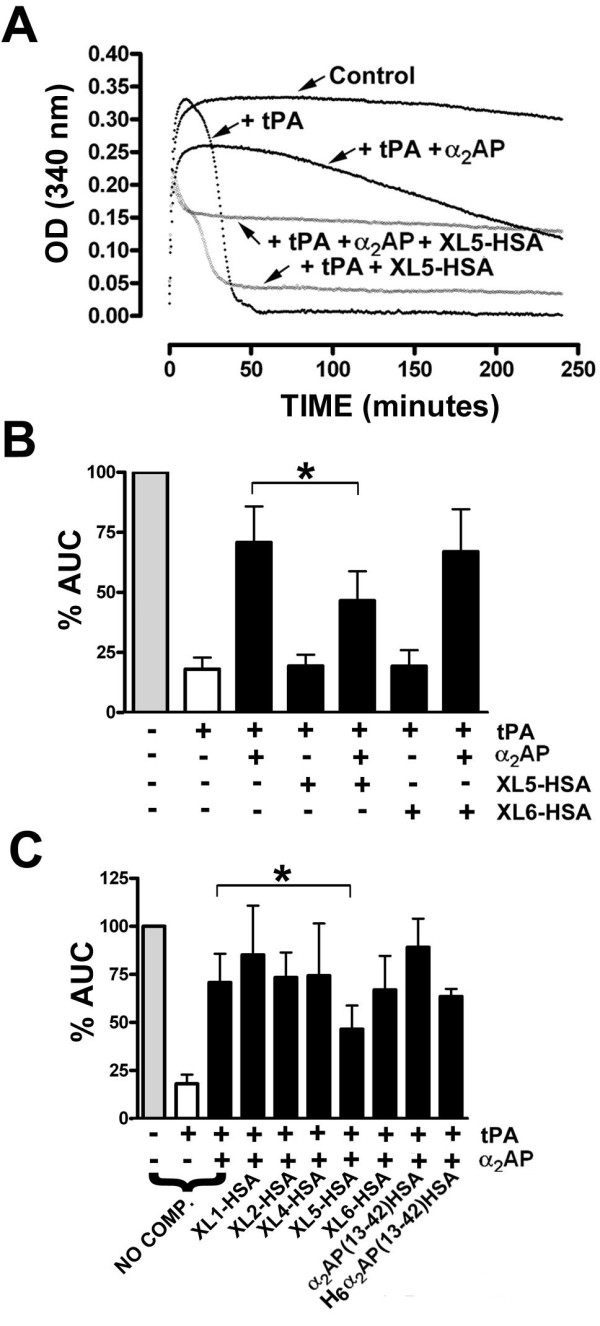
**Effects of α**_**2**_**AP and recombinant HSA-related proteins on plasma clot formation and lysis**. Clot formation and lysis were followed by monitoring turbidity (absorbance at 340 nm) every 30 seconds for 4 hours using a plate reader, of clots formed using diluted α_2_AP-deficient plasma, recalcified with 5 mM CaCl_2 _and supplemented with both 5 nM thrombin and 0.125 nM tPA, and taking the area under the turbidity versus time curve (AUC). Reactions were carried out with or without addition of 1 μM α_2_AP and/or 20 μM fusion protein. (A) shows results of a single representative experiment. (B) shows quantification of turbidity plots for reactions similar to those shown in A, in the presence or absence of XL5-HSA or XL6-HSA competitor proteins; reaction components are indicated (+ or -) below the graph. The mean ± SD of 5-14 determinations is shown. AUC values were normalized, taking the no tPA, no α_2_AP condition as 100% (grey bar); white (open) bars correspond to tPA only condition. (C) shows similar results to panel B for additional competitor proteins identified below the graph. As in panel B, the mean ± SD of 5-14 determinations is shown.

α_2_AP attenuates the ability of tPA to lyse fibrin clots by inhibiting plasmin generated by tPA-mediated activation of plasminogen. This property can be demonstrated *in vitro *using α_2_AP-deficient plasma. As shown in Figure 5A, clots formed in the absence of tPA and α_2_AP were stable for > 4 hours, whereas the addition of tPA lead to complete lysis within 60 minutes. Restoration of α_2_AP to its physiological concentration of 1 μM restored approximately 70% of the area under the turbidity curve. The ability of the HSA-related fusion proteins to compete for the α_2_AP-mediated lysis attenuation was tested by addition of 20 μM fusion proteins. Although at this excess, some changes in the shape of the turbidity plot in the presence of fusion protein and the absence of fusion protein could be noted (Figure 5A), the area under the curve (AUC) of the turbidity curve did not differ from baseline, as exemplified for XL5-HSA and XL6-HSA in Figure 5B. XL5-HSA significantly reduced the α_2_AP-mediated attenuation of clot lysis, shifting the AUC from 70.8 ± 14% of maximal clotting to 40.5 ± 2.9% (Figure 5B); in contrast XL6-HSA did not compete for the α_2_AP effect. Extension of these comparisons to the complete set of recombinant fusion proteins showed that only XL5-HSA and H_6_α_2_AP(13-42)-HSA, under the conditions employed, reduced the α_2_AP effect, and that only the XL5-HSA reduction was statistically significant.

### Fusion protein XL5-HSA is retained in experimental thrombi in mice and rabbits

**Figure 6:**
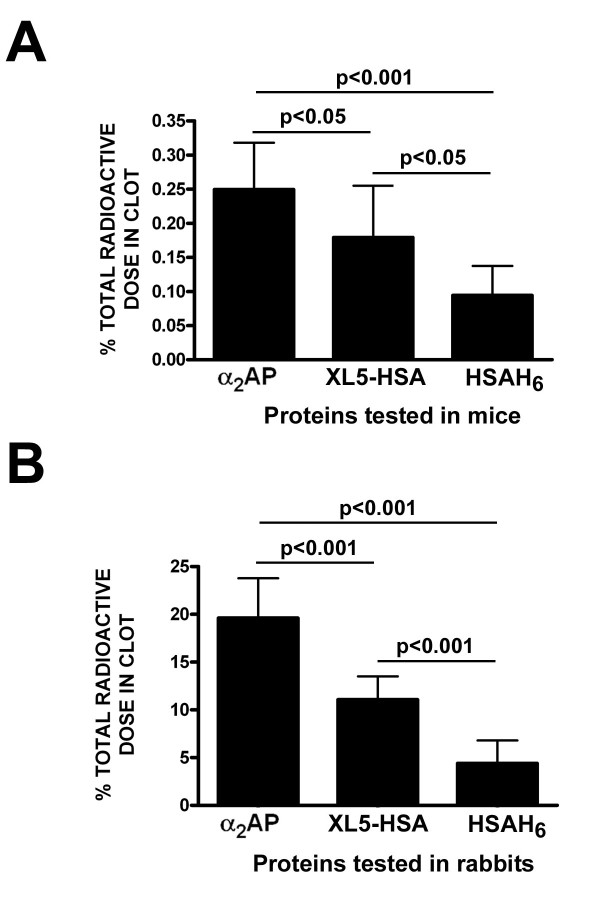
**Retention of radiolabeled proteins in experimental thrombi formed in mice or rabbits**. (A) The radioactivity remaining in clots induced by surgical, topical application of 10% ferric chloride to the *vena cava *of anesthetized mice is shown, as a fraction of the total radioactive protein dose injected, divided by the weight of the clot. Injected radiolabeled proteins are identified on the × axis. The mean ± SD of 12 determinations is shown. (B) The radioactivity remaining in rabbit jugular vein clots formed in clamped-off vessels *in situ *for 30 minutes in the anesthetized animal, then exposed to circulating blood for 60 minutes, prior to clot recovery and γ-counting is shown, expressed as in Figure 6A. The extent (p < 0.001, p < 0.05) of statistical significance is shown for the data sets linked by horizontal lines.

Having obtained several lines of evidence that fusion protein XL5-HSA was the most effective FXIIIa substrate of the recombinant proteins tested, we next asked whether, like α_2_AP, it was capable of being retained within whole blood clots *in vivo*. As shown in Figure 6A, when equal doses of radiolabeled α_2_AP, XL5-HSA, and HSAH_6 _were injected into mice that formed experimental thrombi in the *vena cava *due to ferric chloride administration, both α_2_AP and XL5-HSA were retained within clots to a significantly greater extent than HSAH_6_. The retention of α_2_AP on a clot weight basis, was also significantly greater than that of XL5-HSA.

Similar results were obtained using a larger, rabbit experimental model in which the radiolabeled proteins were introduced, with a coagulation activator, into a whole blood segment within the isolated rabbit jugular vein. As shown in Figure 6B, the three proteins were found to become incorporated into the clot with the same relative efficacy as in the murine model; both α_2_AP and XL5-HSA were incorporated to a greater extent than HSAH_6_. However, in common with the murine results, the XL5-HSA tracer incorporation was significantly less than that of α_2_AP.

### Fusion protein XL5-HSA is retained in a urea-sensitive manner in experimental thrombi in rabbits

**Figure 7:**
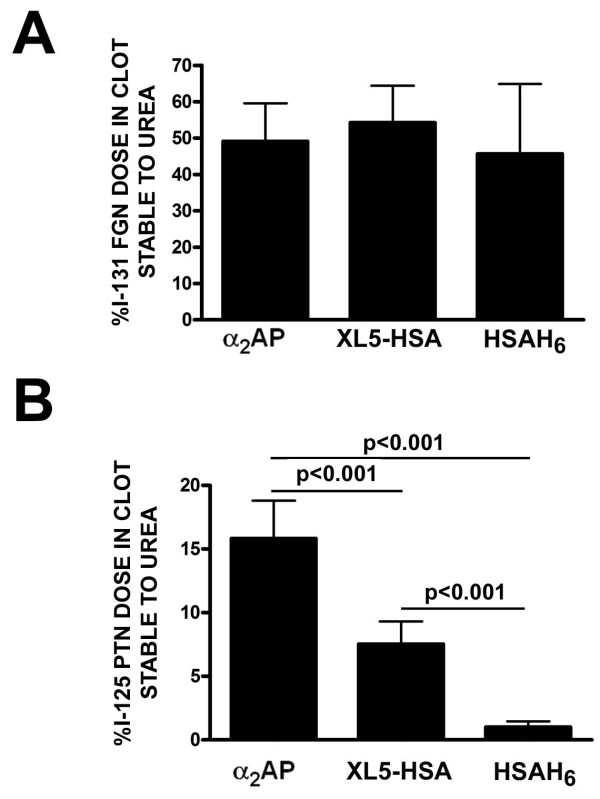
**Retention of radiolabeled proteins in experimental thrombi formed in rabbits following extraction in 5.0 M urea**. Rabbits were co-injected with ^131^I-fibrinogen and ^125^I-proteins identified in Figure 6 and jugular vein clots were formed as described in "Methods" Clots were γ-counted at the close of the experiment, and then extracted overnight with 5.0 M urea, the supernatant aspirated, and the clots recounted. The fraction of the total injected radiolabeled protein dose found in the clot after urea extraction is shown for ^131^I-fibrinogen, identified by the co-injected ^125^I protein on the × axis in (A) and for ^125^I-labeled proteins in (B). Differences between ^131^I-fibrinogen among the three groups in (A) are not statistically significant; the extent of statistically significant differences between groups is shown for the data sets linked by horizontal lines in (B). The mean ± SD of 12 determinations is shown.

In the larger animal model, the rabbit, the greater physical size of the clot permitted its manipulation, unlike with the much smaller murine thrombi. Prolonged stasis and the direct introduction of tissue factor activator into the isolated vessel strongly favoured thrombus formation in the rabbit model, as compared to the localized application of indirectly procoagulant ferric chloride to a localized area of a much smaller vessel in the mouse. Accordingly, clot weights from rabbits ranged from 21.4 to 54.1 mg versus 5.1 to 11.4 mg in mice. This greater size permitted clot extraction in 5.0 M urea and determination of the fraction of clot-bound radioactivity that was insensitive to urea, a hallmark of cross-linked protein clot incorporation. In these experiments, the three ^125^I-labeled proteins were each separately co-administered with ^131^I-labeled fibrinogen. As shown in Figure 7A, approximately 50% of injected fibrinogen was clot-associated in a urea-insensitive manner; there were no differences between treatment groups, as expected because tracer-level doses of the recombinant proteins were employed. While significantly greater amounts of α_2_AP remained clot-bound after urea extraction than XL5-HSA (15.8 ± 2.8% of injected dose versus 7.52 ± 1.7%) (Figure 7B), this represented 15- and 7.5-fold greater urea-insensitive retention than for HSAH_6 _(1.00 ± 0.43%).

## Discussion

In this study, we sought to increase the stability and activity of HSA fusion proteins containing short N-terminal extensions that are substrates for transglutamination by FXIIIa. Our long-term goal was to produce well-tolerated therapeutic proteins for injection, with the long circulatory half-life of albumin, and the ability to compete the cross-linking of α_2_AP to fibrin in thrombi. Replacing active α_2_AP in the clot with a protein incapable of inhibiting plasmin could render pathological clots easier to dissolve. Others have suggested that chemically or mutationally inactivated α_2_AP could serve this purpose [22,23], while we have focused on the albumin fusion strategy [19], due to the ease of large-scale production of recombinant albumin [8]. In this study, we built chimeric albumin fusion proteins modelled on α_2_AP(13-42)-HSA, seeking to eliminate the partial proteolysis of this prototype, but retain and enhance its ability to be cross-linked by FXIIIa. Our approaches included shortening the natural α_2_AP-derived cross-linking site, replacing it with either of two FXIIIa highly active substrate sequences identified by phage display, and capping all three novel chimerae with hexahistidine tags.

Substituting the sequence DQMMLPWPAVTL for α_2_AP(13-42), in fusion protein XL5-HSA, was found to be the most effective approach. This sequence had been reported to be a highly favourable substrate for FXIIIa-mediated transglutamination, when expressed fused to a phage coat protein or to glutathione sulfotransferase (GST) (designated F11 in [26]). When expressed fused to HSA, it yielded the candidate protein most rapidly and efficiently cross-linked to BPA, and the most effective competitor of α_2_AP crosslinking, both with small substrates and in competition of α_2_AP's *in vitro *antifibrinolytic effect. The molecular context of the fusion partner was relevant, since sequence WQHKIDLPYNGA, identical to sequence F28 in [26], except for substitution of protease-susceptible R for P at position 8, appeared to be much less active in fusion protein XL6-HSA than in the GST context. Similarly, α_2_AP(13-23)K24A, although shown to be effectively crosslinked by FXIIIa as α_2_AP(13-23) or α_2_AP(13-24) free peptides [33-35], was ineffective at competing α_2_AP crosslinking to fibrin in fusion protein XL4-HSA. Although our avoidance of Lys or Arg residues in the fusion motifs was successful in avoiding degradation in each case, only XL5-HSA was clearly superior not only to XL4-HSA or XL6-HSA but also to α_2_AP(13-42)-HSA.

Although the requirements for optimal substrates of FXIIIa have not been precisely defined, they are thought to involve positioning of a reactive glutamine residue within a highly flexible sequence [36]. Fusion proteins XL4-, XL5-, and XL6-HSA all contained a reactive glutamine at position 2, analogous to Q14, the most reactive glutamine in the native α_2_AP sequence. Q14 is more effectively cross-linked by FXIIIa in the major form of α_2_AP that circulates in plasma, Asn-α_2_AP, than in the minor precursor form, Met-α_2_AP, which retains residues 1-12 [37]. Our finding that positioning a hexahistidine tag N-terminal to the FXIIIa substrate motif reduced its activity in fusion proteins XL1- and XL2-HSA was consistent both with this natural example and with the general concept of flexibility of the reactive glutamine.

Previously, we found that α_2_AP(13-42)-HSA was no more likely to be present *in vivo *in the rabbit jugular vein model of thrombosis than recombinant HSA [19]. In contrast, XL5-HSA was retained in thrombi *in vivo *to a significantly greater extent than recombinant HSA in both rabbit and murine models. Its degree of retention was less than that of human α_2_AP in both cases, suggesting that there is still room for improvement in defining an optimal sequence to render HSA efficiently cross-linked by factor XIIIa. Nevertheless, this is the first demonstration of targeting of an inactive carrier protein to *in vivo *clots by an attached transamidation substrate motif, although, as described below, others have shown in vivo clot association of transamidation substrate motif peptides [35] and peptide-contrast agent conjugates [33,34]

The insolubility of cross-linked fibrin in 5.0 M urea is a property that has been known for many years, and indeed is used clinically to diagnose FXIII deficiency [38]. Using this property, we showed that *in vivo *clot-associated XL5-HSA is urea-insoluble, like the majority of bound fibrin or α_2_AP, but unlike the majority of bound HSA. Although much indirect or *in vitro *evidence supports the importance of α_2_AP cross-linking to fibrin with respect to clot resistance to fibrinolysis [22,23,39,40], this is to our knowledge the first demonstration of cross-linking of either α_2_AP protein or an engineered polypeptide into *in vivo *thrombi. Other groups have used peptides in this regard, but predominantly in an analytical or imaging mode distinct from long-term goal of reducing thrombus size using cross-linkable proteins. Robinson *et al*. showed that biotinylated α_2_AP (13-24) peptide became cross-linked to human thrombi embolized into the lungs of ferrets and mice when infused *in vivo *[35]. Jaffer *et al*. employed an α_2_AP (13-24) peptide linked to a near infrared fluorochrome to image murine thrombi formed following ferric chloride treatment of the femoral vessels [34], while Miserus *et al.*, used a similar α_2_AP (13-23) peptide conjugated to a gadolinium-containing contrast agent to visualize murine carotid artery ferric chloride-induced thrombi [33]. While these peptides and peptide-chemical conjugates show great promise for future improved detection of thrombi and stratification of patients, peptides are typically cleared from the circulation with extremely rapid pharmacokinetics. That fusion protein XL5-HSA was found to localize in *in vivo *thrombi to a greater extent than unmodified recombinant HSA suggests not only its superiority over our previously described α_2_AP (13-42)-HSA chimera, but also its potential utility in being incorporated as a "Trojan horse" into thrombi, and making them more susceptible to natural or pharmacological thrombolysis.

## Conclusions

Recombinant HSA with a DQMMLPWPAVTLG_4_Y N-terminal extension (XL5-HSA) was more active as a substrate for FXIIIa-mediated cross-linking to either artificial or natural transglutamination partners than other candidate motifs of identical or longer length, with or without an N-terminal His tag. XL5-HSA, unlike α_2_AP(13-42)-HSA or H_6_α_2_AP(13-42)-HSA, was not subject to detectable proteolysis by *P. pastoris*. Of the proteins tested, XL5-HSA was the most effective competitor of α_2_AP-mediated resistance of fibrin clots to lysis. In contrast to previous results with α_2_AP(13-42)-HSA, radiolabeled tracer XL5-HSA was found to a greater extent in experimentally-induced intravascular clots in both murine and rabbit *in vivo *venous thrombosis models; importantly, the majority of clot-associated XL5-HSA and plasma-derived α_2_AP, but not recombinant HSA, was shown to be insoluble to 5.0 M urea, suggesting covalent cross-linking. Our results suggest that fusion protein XL5-HSA has been sufficiently optimized over prototype α_2_AP(13-42)-HSA to warrant *in vivo *testing as a potential protein drug rendering thrombi more susceptible to natural or pharmacological clot lysis.

## Authors' contributions

WPS conceived of the study, secured competitive funding, directed experiments, and wrote the manuscript. LJE-S performed all *in vitro *and *in vivo *experiments and developed and refined experimental protocols. Both authors participated in editing and revising the manuscript. Both authors read and approved the final manuscript.
